# 2,4,6-Tri­amino-1,3,5-triazin-1-ium 3-(prop-2-eno­yloxy)propano­ate acrylic acid monosolvate monohydrate

**DOI:** 10.1107/S1600536813009999

**Published:** 2013-04-17

**Authors:** V. Sangeetha, N. Kanagathara, G. Chakkaravarthi, M. K. Marchewka, G. Anbalagan

**Affiliations:** a Department of Physics, D.G. Vaishnav College, Chennai 600 106, India; b Department of Physics, Vel Tech Multi Tech Dr. Rangarajan and Dr. Sakunthala Eng. College, Chennai 600 062, India; c Department of Physics, CPCL Polytechnic College, Chennai 600 068, India; d Institute of Low Temperature and Structure Research, Polish Academy of Sciences, 50-950 Wrocław, 2, PO Box 937, Poland; e Department of Physics, Presidency College, Chennai 600 005, India

## Abstract

The asymmetric unit of the title salt, C_3_H_7_N_6_^+^·C_6_H_7_O_4_^−^·C_3_H_4_O_2_·H_2_O, contains a 2,4,6-tri­amino-1,3,5-triazin-1-ium cation, a 3-(prop-2-eno­yloxy)propano­ate anion and acrylic acid and water solvent mol­ecules in a 1:1:1:1 ratio and with each species in a general position. In the crystal, the components are linked into a supra­molecular layer in the *bc* plane *via* a combination of O—H⋯O, N—H⋯N and N—H⋯O hydrogen bonding. The crystal studied was a non-merohedral twin, the minor component contribution being approximately 26%.

## Related literature

For general background to melamine derivatives, see: Krische & Lehn, (2000 ▶). For related structures, see: Kanagathara *et al.* (2012 ▶); Wang *et al.* (2007 ▶).
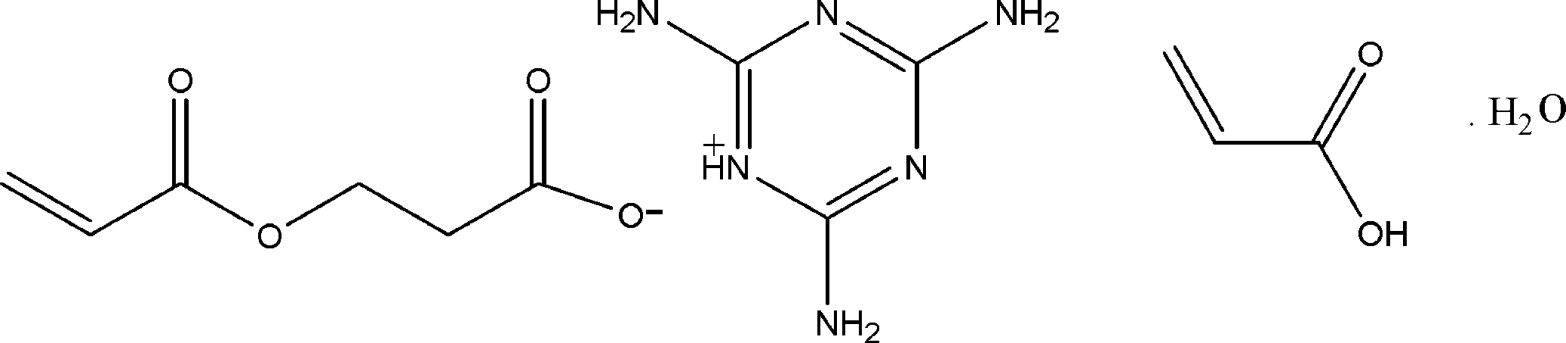


## Experimental

### 

#### Crystal data

C_3_H_7_N_6_^+^·C_6_H_7_O_4_^−^·C_3_H_4_O_2_·H_2_O
*M* *_r_* = 360.34
Triclinic, 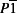

*a* = 4.84800 (1) Å
*b* = 12.4200 (2) Å
*c* = 14.8850 (3) Å
α = 101.010 (1)°
β = 92.652 (1)°
γ = 94.117 (1)°
*V* = 875.84 (3) Å^3^
*Z* = 2
Mo *K*α radiation
μ = 0.11 mm^−1^
*T* = 295 K
0.30 × 0.26 × 0.24 mm

#### Data collection

Bruker Kappa APEXII CCD diffractometer
Absorption correction: multi-scan (*SADABS*; Sheldrick, 1996 ▶) *T*_min_ = 0.967, *T*_max_ = 0.973
14152 measured reflections
14152 independent reflections
10635 reflections with *I* > 2σ(*I*)
*R*_int_ = 0.000

#### Refinement

*R*[*F* ^2^ > 2σ(*F* ^2^)] = 0.059
*wR*(*F* ^2^) = 0.204
*S* = 1.07
14152 reflections
264 parameters
3 restraints
H atoms treated by a mixture of independent and constrained refinement
Δρ_max_ = 0.40 e Å^−3^
Δρ_min_ = −0.25 e Å^−3^

### 

Data collection: *APEX2* (Bruker, 2004 ▶); cell refinement: *SAINT* (Bruker, 2004 ▶); data reduction: *SAINT*; program(s) used to solve structure: *SHELXS97* (Sheldrick, 2008 ▶); program(s) used to refine structure: *SHELXL97* (Sheldrick, 2008 ▶); molecular graphics: *PLATON* (Spek, 2009 ▶); software used to prepare material for publication: *SHELXL97*.

## Supplementary Material

Crystal structure: contains datablock(s) I, global. DOI: 10.1107/S1600536813009999/tk5217sup1.cif

Structure factors: contains datablock(s) I. DOI: 10.1107/S1600536813009999/tk5217Isup2.hkl

Supplementary material file. DOI: 10.1107/S1600536813009999/tk5217Isup3.cml

Additional supplementary materials:  crystallographic information; 3D view; checkCIF report

## Figures and Tables

**Table 1:** Hydrogen-bond geometry (Å, °)

*D*—H⋯*A*	*D*—H	H⋯*A*	*D*⋯*A*	*D*—H⋯*A*
O7—H7*A*⋯O4	0.84 (1)	2.05 (2)	2.804 (2)	149 (3)
O1—H1⋯O3^i^	0.82	1.77	2.5872 (17)	171
N1—H1*A*⋯O3^ii^	0.89 (1)	1.90 (1)	2.7829 (17)	175 (2)
N4—H4*C*⋯N2^i^	0.922 (19)	2.08 (2)	2.995 (2)	175 (16)
N4—H4*D*⋯O3^ii^	0.953 (15)	2.494 (16)	3.295 (2)	142 (12)
N4—H4*D*⋯O2^iii^	0.953 (15)	2.172 (15)	2.850 (2)	127 (12)
N5—H5*A*⋯O2^iv^	0.97 (2)	2.03 (2)	3.001 (2)	175 (17)
N5—H5*B*⋯O7^v^	0.898 (18)	2.031 (18)	2.875 (2)	156 (14)
N6—H6*B*⋯N3^vi^	0.88 (2)	2.17 (2)	3.039 (2)	169 (17)
O7—H7*B*⋯O6^iv^	0.84 (1)	2.17 (2)	2.977 (3)	161 (4)

Symmetry codes: (i) 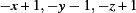; (ii) 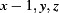; (iii) 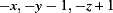; (iv) 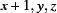; (v) 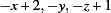; (vi) 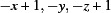.

**Fig. 1.**
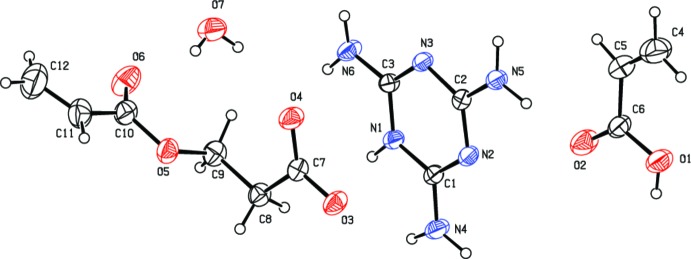
The molecular structure of the constituents of (I), showing atom labels and 30% probability displacement ellipsoids for non-H atoms.

**Fig. 2.**
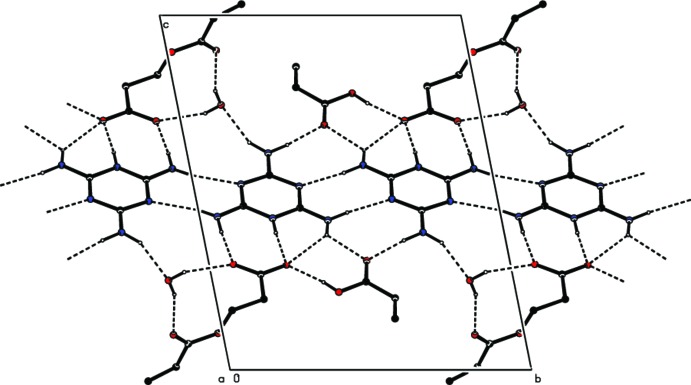
A view of the packing of (I) in projection down the a axis. Hydrogen bonds are shown as dashed lines.

## supplementary crystallographic information

### Comment

Melamine and its derivatives can develop well defined non-covalent supramolecular nanoarchitectures *via* multiple hydrogen bonds by self-assembly of components containing complementary arrays of hydrogen-bonding sites (Krische & Lehn, 2000). The geometric parameters of the title compound (I), Fig. 1, are comparable with similar structures (Kanagathara *et al.*, 2012; Wang *et al.*, 2007).

The crystal packing is stabilized by intermolecular O—H···O, N—H···O, N—H···N and C—H···O interactions to form layers in the *b*c-plane, Fig. 2.

### Experimental

Melamine (0.64 g, 5 mmol) and acrylic acid (0.36 g, 5 mmol) were taken in 1:3 ratio. Melamine was dissolved in a hot solution (100 ml) of distilled water. Acrylic acid (1.3911 g, 0.01 mmol) was dissolved in distilled water (5 ml) separately. To the hot solution of melamine, the acrylic acid solution was added slowly, and stirred well for nearly four hours to get a homogeneous solution. Then, the mixture is allowed to evaporate. After several days, transparent crystals suitable for X-ray diffractions were formed.

### Refinement

The C-bound H atoms were geometrically placed (C—H = 0.93–0.97 Å) and refined as riding with *U_iso_*(H) = 1.2*U_eq_*(C). The H atoms bound to O and N atoms were found from difference Fourier maps and refined isotropically, with distance restraints N—H = 0.88±0.01 Å and O—H = 0.82 ±0.01 Å; the hydroxyl-H atom was included in its calculated position with O—H = 0.82 Å. The crystal is a non-merohedral twin and the minor component contributes approximately 26%.

### Crystal data

**Table:**

C_3_H_7_N_6_^+^·C_6_H_7_O_4_^−^·C_3_H_4_O_2_·H_2_O	*Z* = 2
*M**_r_* = 360.34	*F*(000) = 380
Triclinic, *P*1	*D*_x_ = 1.366 Mg m^−^^3^
Hall symbol: -P 1	Mo *K*α radiation, λ = 0.71073 Å
*a* = 4.84800 (1) Å	Cell parameters from 3859 reflections
*b* = 12.4200 (2) Å	θ = 2.0–25.0°
*c* = 14.8850 (3) Å	µ = 0.11 mm^−^^1^
α = 101.010 (1)°	*T* = 295 K
β = 92.652 (1)°	Block, colourless
γ = 94.117 (1)°	0.30 × 0.26 × 0.24 mm
*V* = 875.84 (3) Å^3^	

### Data collection

**Table:**

Bruker Kappa APEXII CCD diffractometer	14152 independent reflections
Radiation source: fine-focus sealed tube	10635 reflections with *I* > 2σ(*I*)
Graphite monochromator	*R*_int_ = 0.000
ω and φ scan	θ_max_ = 25.0°, θ_min_ = 2.0°
Absorption correction: multi-scan (*SADABS*; Sheldrick, 1996)	*h* = −5→5
*T*_min_ = 0.967, *T*_max_ = 0.973	*k* = −14→14
14152 measured reflections	*l* = −17→17

### Refinement

**Table:**

Refinement on *F*^2^	Secondary atom site location: difference Fourier map
Least-squares matrix: full	Hydrogen site location: inferred from neighbouring sites
*R*[*F*^2^ > 2σ(*F*^2^)] = 0.059	H atoms treated by a mixture of independent and constrained refinement
*wR*(*F*^2^) = 0.204	*w* = 1/[σ^2^(*F*_o_^2^) + (0.1084*P*)^2^ + 0.3527*P*] where *P* = (*F*_o_^2^ + 2*F*_c_^2^)/3
*S* = 1.07	(Δ/σ)_max_ < 0.001
14152 reflections	Δρ_max_ = 0.40 e Å^−^^3^
264 parameters	Δρ_min_ = −0.25 e Å^−^^3^
3 restraints	Extinction correction: *SHELXL97* (Sheldrick, 2008), Fc^*^=kFc[1+0.001xFc^2^λ^3^/sin(2θ)]^-1/4^
Primary atom site location: structure-invariant direct methods	Extinction coefficient: 0.011 (2)

### Special details

**Table:**

Geometry. All e.s.d.'s (except the e.s.d. in the dihedral angle between two l.s. planes) are estimated using the full covariance matrix. The cell e.s.d.'s are taken into account individually in the estimation of e.s.d.'s in distances, angles and torsion angles; correlations between e.s.d.'s in cell parameters are only used when they are defined by crystal symmetry. An approximate (isotropic) treatment of cell e.s.d.'s is used for estimating e.s.d.'s involving l.s. planes.
Refinement. Refinement of *F*^2^ against ALL reflections. The weighted *R*-factor *wR* and goodness of fit *S* are based on *F*^2^, conventional *R*-factors *R* are based on *F*, with *F* set to zero for negative *F*^2^. The threshold expression of *F*^2^ > σ(*F*^2^) is used only for calculating *R*-factors(gt) *etc*. and is not relevant to the choice of reflections for refinement. *R*-factors based on *F*^2^ are statistically about twice as large as those based on *F*, and *R*- factors based on ALL data will be even larger.

### Fractional atomic coordinates and isotropic or equivalent isotropic displacement parameters (Å^2^)

**Table:**

	*x*	*y*	*z*	*U*_iso_*/*U*_eq_	
C1	0.3604 (3)	−0.33906 (13)	0.54291 (10)	0.0388 (4)	
C2	0.6285 (3)	−0.25897 (13)	0.45046 (10)	0.0387 (4)	
C3	0.3461 (3)	−0.15225 (13)	0.53714 (10)	0.0376 (4)	
C4	0.5541 (5)	−0.4187 (2)	0.14464 (17)	0.0881 (7)	
H4A	0.6272	−0.4866	0.1297	0.106*	
H4B	0.6184	−0.3606	0.1179	0.106*	
C5	0.3624 (4)	−0.40507 (17)	0.20271 (14)	0.0658 (6)	
H5	0.2942	−0.3361	0.2160	0.079*	
C6	0.2452 (4)	−0.49050 (15)	0.24902 (13)	0.0525 (5)	
C7	0.7769 (3)	−0.16472 (14)	0.73027 (11)	0.0412 (4)	
C8	0.5604 (3)	−0.15879 (14)	0.80048 (11)	0.0461 (4)	
H8A	0.4012	−0.2087	0.7746	0.055*	
H8B	0.6352	−0.1844	0.8534	0.055*	
C9	0.4644 (3)	−0.04632 (15)	0.83200 (12)	0.0501 (5)	
H9A	0.4362	−0.0105	0.7802	0.060*	
H9B	0.2907	−0.0516	0.8613	0.060*	
C10	0.6404 (4)	0.12157 (16)	0.92622 (12)	0.0535 (5)	
C11	0.8622 (4)	0.17721 (19)	0.99161 (14)	0.0669 (6)	
H11	1.0107	0.1383	1.0049	0.080*	
C12	0.8592 (6)	0.2792 (2)	1.03178 (18)	0.1037 (9)	
H12A	0.7122	0.3192	1.0192	0.124*	
H12B	1.0041	0.3123	1.0732	0.124*	
N1	0.2537 (3)	−0.24126 (11)	0.57194 (9)	0.0404 (3)	
H1A	0.128 (3)	−0.2424 (18)	0.6135 (11)	0.074 (6)*	
N2	0.5509 (3)	−0.35026 (11)	0.48202 (9)	0.0421 (3)	
N3	0.5367 (3)	−0.15873 (11)	0.47602 (8)	0.0392 (3)	
N4	0.2644 (3)	−0.42345 (13)	0.57764 (11)	0.0536 (4)	
H4C	0.323 (3)	−0.4922 (17)	0.5560 (12)	0.057 (5)*	
H4D	0.121 (3)	−0.4097 (13)	0.6191 (11)	0.042 (4)*	
N5	0.8195 (3)	−0.26917 (14)	0.38830 (9)	0.0467 (4)	
H5A	0.899 (4)	−0.3378 (19)	0.3654 (14)	0.081 (7)*	
H5B	0.862 (3)	−0.2142 (14)	0.3587 (11)	0.042 (5)*	
N6	0.2362 (3)	−0.05879 (13)	0.56754 (11)	0.0492 (4)	
H6A	0.110 (4)	−0.0621 (15)	0.6107 (13)	0.055 (5)*	
H6B	0.317 (4)	−0.0013 (17)	0.5495 (12)	0.062 (6)*	
O1	0.3317 (3)	−0.58757 (11)	0.21954 (9)	0.0694 (4)	
H1	0.2598	−0.6320	0.2475	0.104*	
O2	0.0843 (3)	−0.47429 (11)	0.30853 (10)	0.0718 (4)	
O3	0.8711 (2)	−0.25670 (10)	0.70356 (8)	0.0532 (3)	
O4	0.8527 (3)	−0.08094 (10)	0.70184 (8)	0.0563 (3)	
O5	0.6780 (2)	0.01609 (10)	0.89658 (8)	0.0518 (3)	
O6	0.4413 (3)	0.16469 (12)	0.90105 (11)	0.0773 (4)	
O7	1.0138 (4)	0.14515 (14)	0.74741 (12)	0.0742 (4)	
H7A	0.915 (5)	0.0873 (16)	0.724 (2)	0.141 (14)*	
H7B	1.130 (6)	0.135 (4)	0.787 (2)	0.21 (2)*	

### Atomic displacement parameters (Å^2^)

**Table:**

	*U*^11^	*U*^22^	*U*^33^	*U*^12^	*U*^13^	*U*^23^
C1	0.0455 (9)	0.0298 (10)	0.0405 (9)	0.0014 (7)	0.0056 (7)	0.0058 (7)
C2	0.0450 (9)	0.0327 (10)	0.0367 (8)	0.0016 (7)	0.0031 (7)	0.0028 (7)
C3	0.0422 (9)	0.0324 (10)	0.0389 (8)	0.0013 (7)	0.0007 (7)	0.0097 (7)
C4	0.115 (2)	0.0604 (16)	0.0961 (17)	0.0067 (13)	0.0356 (16)	0.0270 (13)
C5	0.0868 (15)	0.0412 (13)	0.0705 (13)	0.0073 (10)	0.0062 (12)	0.0123 (10)
C6	0.0622 (12)	0.0352 (11)	0.0585 (11)	0.0059 (9)	−0.0014 (9)	0.0053 (9)
C7	0.0376 (9)	0.0386 (11)	0.0452 (9)	0.0028 (8)	0.0021 (7)	0.0029 (8)
C8	0.0439 (10)	0.0435 (11)	0.0507 (10)	0.0046 (8)	0.0110 (8)	0.0062 (8)
C9	0.0428 (10)	0.0510 (12)	0.0561 (11)	0.0088 (8)	0.0094 (8)	0.0061 (9)
C10	0.0590 (12)	0.0473 (13)	0.0550 (11)	0.0076 (9)	0.0255 (9)	0.0059 (9)
C11	0.0728 (14)	0.0657 (16)	0.0580 (12)	0.0051 (11)	0.0127 (10)	−0.0007 (11)
C12	0.128 (2)	0.075 (2)	0.0941 (19)	0.0066 (16)	−0.0105 (17)	−0.0134 (15)
N1	0.0478 (8)	0.0316 (8)	0.0426 (7)	0.0014 (6)	0.0086 (7)	0.0090 (6)
N2	0.0524 (8)	0.0292 (8)	0.0444 (8)	0.0025 (6)	0.0112 (6)	0.0048 (6)
N3	0.0469 (8)	0.0289 (8)	0.0430 (8)	0.0043 (6)	0.0098 (6)	0.0076 (6)
N4	0.0719 (11)	0.0323 (9)	0.0606 (10)	0.0060 (8)	0.0244 (9)	0.0134 (7)
N5	0.0638 (10)	0.0354 (9)	0.0443 (8)	0.0083 (7)	0.0221 (7)	0.0100 (7)
N6	0.0562 (10)	0.0324 (9)	0.0628 (10)	0.0106 (7)	0.0243 (8)	0.0117 (7)
O1	0.0963 (11)	0.0413 (9)	0.0760 (9)	0.0149 (7)	0.0341 (8)	0.0146 (6)
O2	0.0886 (10)	0.0496 (9)	0.0828 (10)	0.0199 (7)	0.0371 (8)	0.0139 (7)
O3	0.0568 (7)	0.0376 (8)	0.0657 (8)	0.0089 (6)	0.0222 (6)	0.0053 (6)
O4	0.0680 (8)	0.0412 (8)	0.0633 (8)	0.0059 (6)	0.0269 (6)	0.0132 (6)
O5	0.0562 (8)	0.0462 (8)	0.0506 (7)	0.0119 (6)	0.0054 (6)	0.0005 (6)
O6	0.0733 (10)	0.0545 (10)	0.1014 (12)	0.0216 (7)	0.0028 (8)	0.0034 (8)
O7	0.0988 (13)	0.0528 (10)	0.0763 (10)	0.0053 (9)	0.0210 (10)	0.0227 (8)

### Geometric parameters (Å, º)

**Table:**

C1—N4	1.320 (2)	C8—H8B	0.9700
C1—N2	1.3198 (19)	C9—O5	1.451 (2)
C1—N1	1.356 (2)	C9—H9A	0.9700
C2—N5	1.335 (2)	C9—H9B	0.9700
C2—N2	1.344 (2)	C10—O6	1.215 (2)
C2—N3	1.344 (2)	C10—O5	1.329 (2)
C3—N6	1.321 (2)	C10—C11	1.456 (3)
C3—N3	1.3225 (18)	C11—C12	1.294 (3)
C3—N1	1.364 (2)	C11—H11	0.9300
C4—C5	1.296 (3)	C12—H12A	0.9300
C4—H4A	0.9300	C12—H12B	0.9300
C4—H4B	0.9300	N1—H1A	0.889 (9)
C5—C6	1.467 (3)	N4—H4C	0.922 (19)
C5—H5	0.9300	N4—H4D	0.953 (15)
C6—O2	1.205 (2)	N5—H5A	0.97 (2)
C6—O1	1.308 (2)	N5—H5B	0.898 (18)
C7—O4	1.235 (2)	N6—H6A	0.911 (18)
C7—O3	1.2609 (19)	N6—H6B	0.88 (2)
C7—C8	1.511 (2)	O1—H1	0.8200
C8—C9	1.500 (2)	O7—H7A	0.843 (10)
C8—H8A	0.9700	O7—H7B	0.836 (10)
			
N4—C1—N2	120.99 (15)	O5—C9—H9B	110.3
N4—C1—N1	117.24 (15)	C8—C9—H9B	110.3
N2—C1—N1	121.77 (14)	H9A—C9—H9B	108.5
N5—C2—N2	116.22 (14)	O6—C10—O5	122.83 (18)
N5—C2—N3	117.01 (15)	O6—C10—C11	125.0 (2)
N2—C2—N3	126.76 (14)	O5—C10—C11	112.21 (17)
N6—C3—N3	121.40 (15)	C12—C11—C10	122.1 (2)
N6—C3—N1	116.79 (15)	C12—C11—H11	119.0
N3—C3—N1	121.80 (14)	C10—C11—H11	119.0
C5—C4—H4A	120.0	C11—C12—H12A	120.0
C5—C4—H4B	120.0	C11—C12—H12B	120.0
H4A—C4—H4B	120.0	H12A—C12—H12B	120.0
C4—C5—C6	124.8 (2)	C1—N1—C3	119.00 (13)
C4—C5—H5	117.6	C1—N1—H1A	114.6 (14)
C6—C5—H5	117.6	C3—N1—H1A	126.4 (14)
O2—C6—O1	122.40 (17)	C1—N2—C2	115.50 (13)
O2—C6—C5	124.07 (18)	C3—N3—C2	115.15 (13)
O1—C6—C5	113.53 (17)	C1—N4—H4C	119.3 (11)
O4—C7—O3	123.29 (15)	C1—N4—H4D	116.1 (9)
O4—C7—C8	119.37 (15)	H4C—N4—H4D	124.2 (15)
O3—C7—C8	117.35 (15)	C2—N5—H5A	123.5 (12)
C9—C8—C7	114.76 (15)	C2—N5—H5B	120.4 (10)
C9—C8—H8A	108.6	H5A—N5—H5B	115.5 (16)
C7—C8—H8A	108.6	C3—N6—H6A	114.9 (11)
C9—C8—H8B	108.6	C3—N6—H6B	114.1 (12)
C7—C8—H8B	108.6	H6A—N6—H6B	130.1 (17)
H8A—C8—H8B	107.6	C6—O1—H1	109.5
O5—C9—C8	107.26 (13)	C10—O5—C9	115.98 (13)
O5—C9—H9A	110.3	H7A—O7—H7B	112 (4)
C8—C9—H9A	110.3		
			
C4—C5—C6—O2	173.4 (2)	N4—C1—N2—C2	178.84 (15)
C4—C5—C6—O1	−6.6 (3)	N1—C1—N2—C2	−0.7 (2)
O4—C7—C8—C9	−2.2 (2)	N5—C2—N2—C1	−179.50 (14)
O3—C7—C8—C9	177.86 (15)	N3—C2—N2—C1	1.6 (2)
C7—C8—C9—O5	−77.34 (18)	N6—C3—N3—C2	−179.36 (15)
O6—C10—C11—C12	2.7 (3)	N1—C3—N3—C2	0.8 (2)
O5—C10—C11—C12	−177.0 (2)	N5—C2—N3—C3	179.48 (14)
N4—C1—N1—C3	−179.52 (15)	N2—C2—N3—C3	−1.6 (2)
N2—C1—N1—C3	0.0 (2)	O6—C10—O5—C9	−0.5 (2)
N6—C3—N1—C1	−179.95 (14)	C11—C10—O5—C9	179.17 (14)
N3—C3—N1—C1	−0.1 (2)	C8—C9—O5—C10	174.42 (13)

### Hydrogen-bond geometry (Å, º)

**Table:**

*D*—H···*A*	*D*—H	H···*A*	*D*···*A*	*D*—H···*A*
O7—H7*A*···O4	0.84 (1)	2.05 (2)	2.804 (2)	149 (3)
O1—H1···O3^i^	0.82	1.77	2.5872 (17)	171
N1—H1*A*···O3^ii^	0.89 (1)	1.90 (1)	2.7829 (17)	175 (2)
N4—H4*C*···N2^i^	0.922 (19)	2.08 (2)	2.995 (2)	175 (16)
N4—H4*D*···O3^ii^	0.953 (15)	2.494 (16)	3.295 (2)	142 (12)
N4—H4*D*···O2^iii^	0.953 (15)	2.172 (15)	2.850 (2)	127 (12)
N5—H5*A*···O2^iv^	0.97 (2)	2.03 (2)	3.001 (2)	175 (17)
N5—H5*B*···O7^v^	0.898 (18)	2.031 (18)	2.875 (2)	156 (14)
N6—H6*B*···N3^vi^	0.88 (2)	2.17 (2)	3.039 (2)	169 (17)
O7—H7*B*···O6^iv^	0.84 (1)	2.17 (2)	2.977 (3)	161 (4)

Symmetry codes: (i) −*x*+1, −*y*−1, −*z*+1; (ii) *x*−1, *y*, *z*; (iii) −*x*, −*y*−1, −*z*+1; (iv) *x*+1, *y*, *z*; (v) −*x*+2, −*y*, −*z*+1; (vi) −*x*+1, −*y*, −*z*+1.
